# IL17RA and IL21R polymorphisms influence type 1 diabetes predisposition and autoimmune phenotypes

**DOI:** 10.3389/fendo.2025.1620509

**Published:** 2025-09-04

**Authors:** Cintia Semzezem, Karla Fabiana Brasil Gomes, Aritania Sousa Santos, Pauline Brochet, Lindiane Gomes Crisostomo, Marcia Regina Soares Correia, Amanda Farage Frade-Barros, Luciano Abreu Brito, Maria Rita Passos-Bueno, Christophe Chevillard, Edecio Cunha-Neto, Maria Elizabeth Rossi da Silva

**Affiliations:** ^1^ Laboratório de Carboidratos e Radioimunoensaios (LIM-18) do Hospital das Clínicas da Faculdade de Medicina da Universidade de São Paulo, São Paulo, Brazil; ^2^ French National Institute for Health and Medical Research (INSERM), Unité Mixte de Recherche (UMR) U1090, Aix Marseille University (AMU), Theories and Approaches of Genomic Complexity (TAGC), MarMaRa institute, Marseille, France; ^3^ Heart Institute, Laboratory of Clinical Immunology and Allergy-LIM60- Instituto do Coração (INCOR), Hospital das Clínicas da Faculdade de Medicina da Universidade de São Paulo, Institute for Investigation in Immunology-iii/National Institute on Science and Technology (INCT), São Paulo, Brazil; ^4^ Departamento de Genética e Biologia Evolutiva, Instituto deBiociências da Universidade de São Paulo, São Paulo, Brazil

**Keywords:** IL-17RA, IL-21R, type 1 diabetes, islet autoantibodies, extra-pancreatic autoantibodies

## Abstract

**Introduction:**

Although interleukin receptors have been implicated in various autoimmune diseases, their role in type 1 diabetes (T1D) remains underexplored and occasionally inconsistent. To evaluate the impact of polymorphisms in genes encoding the interleukin receptors IL-21R and IL-17RA on T1D susceptibility and other autoimmune manifestations, we analyzed 639 patients with T1D and 653 healthy controls.

**Methods:**

Selected variants in IL17RA (n=4), IL21R (n=4), were genotyped using the VeraCode GoldenGate assay (Illumina, USA). Autoantibodies were assessed by radioimmunoassay, ELISA, and a radiolabeled iodine receptor assay.

**Results:**

Two IL17RA variants were significantly associated with T1D: the rs2241049G allele was linked to increased susceptibility (OR=1.42; *p*=0.005), whereas the rs879577A allele was related to protection (OR=0.61; *p*=0.021) and a reduced frequency of anti-tyrosine phosphatase (anti-IA2) autoantibody (OR= 0.52; *p*=0.010). Additionally, the rs5748863G allele was also associated with a lower frequency of anti-IA2 positivity (OR=0.52; *p*=0.010). Among IL21R variants, only rs7199138C was associated with an increased risk of T1D (OR=1.33; *p*=0.018). Moreover, rs2214537G and rs2285452A were linked to a reduced frequency of anti-parietal cell (OR=0.24; *p*<0.001) and anti-endomysium (OR=0.17; *p*=0.025) autoantibodies, respectively. In contrast, rs2285452A and rs3093315T were related to a higher frequency of anti-thyroperoxidase (OR=2.38; *p*=0.028) and TSH receptor (TRAb) autoantibodies (OR=5.90; *p*=0.024), respectively.

**Discussion:**

These findings suggest that polymorphisms in IL17RA and IL21R genes may contribute to T1D pathogenesis and modulate the presence of pancreatic and extra-pancreatic autoantibodies.

## Introduction

Type 1 diabetes (T1D) is a chronic autoimmune disease with a multifactorial etiology involving environmental triggers, genetic susceptibility, and immunological dysregulation that culminate in the destruction of pancreatic β-cells (1, 2). It is characterized by the presence of high-risk HLA-DR3 and DR4 alleles and islet-specific autoantibodies, which typically appear before clinical onset and reflect the underlying autoimmune process (3). Moreover, T1D is frequently associated with other autoimmune diseases, often accompanied by organ-specific autoantibodies and shared genetic determinants (1, 2).

Recent studies have highlighted the involvement of the Th17 pathway in the immunopathogenesis of T1D, acting independently of the classical Th1 and Th2 pathways. Th17 cells secrete proinflammatory cytokines such as Interleukins IL-17A, IL-21, IL-22, tumor necrosis factor (TNF)-α, and IL-6 (4–6), playing pivotal roles in host defense and in the pathogenesis of autoimmune diseases including autoimmune thyroid disease (7) systemic lupus erythematosus and multiple sclerosis (5, 6, 8–10). Increased circulating levels of IL-17A (11) and mRNA expression of IL-17A have been observed in circulating memory CD4^+^ T cells from children with new-onset T1D (12); furthermore, local IL-17 production has been detected in pancreatic islets around the time of disease onset (13) and human β-cells were shown to be susceptible to IL-17A–induced apoptosis (12). On the other hand, serum levels of IL-17 or IL-23 – a cytokine known to maintain Th17 cells (14, 15), as well as genetic variants in other cytokine genes within the Th17 pathway, such as IL-17 (14), IL-21 (16), and IL-27 (17), were found by our group not to influence T1D susceptibility, clinical phenotype or presence of autoantibodies. In addition, two IL-23A gene variants (rs11171806 and rs2066808; GG haplotype) were associated with protection against T1D in the Brazilian population (15).

In animal models of autoimmune diabetes, IL-17A and Th17 cells have been implicated in disease progression. Interventions with anti-IL-17 antibodies or IL-2 - a negative regulator of Th17 responses—prevented insulitis (18) and remitted diabetes (19), whereas IL-23 administration, which enhances IL-17 and TNF-α production, exacerbated β-cell damage (20). Additionally, the synergistic effect of IL-17A with IL-21 and IFN-γ has been reported to promote diabetes development (21). Furthermore, IL-21 overexpression in pancreatic β-cells led to increased expression of inflammatory mediators such as IL-17A, IL-17F, IFN-γ, MCP-1, MCP-2, and IP-10, whereas IL-21R deficiency conferred protection against insulitis and diabetes in NOD mice (22).

However, some studies have challenged the pathogenic role of Th17 cells in experimental autoimmune diabetes models under specific circumstances (23–26). This data is in line with our previous findings in Brazilian patients with T1D (14–17) that suggested that genetic polymorphisms in cytokines of the Th17 inflammatory pathway may exert a neutral or even protective effect on T1D susceptibility. Few studies evaluated the role of IL17RA and IL21R in human T1D pathogenesis. In prior researches involving Brazilian patients with recent-onset T1D —up to six months after diagnosis, our group reported reduced surface expression of IL-17RA in CD3+CD4+ T cells (14). In this paper, we show that, likewise, IL-21R surface expression is also reduced in CD3+CD4+ T cells from recent-onset T1D patients.

Based on these observations, we hypothesized that genetic variants in the IL17RA and IL21R genes might modulate IL-17 and IL-21 signaling, thereby contributing to T1D pathogenesis. To investigate this, we selected tag single nucleotide polymorphisms (SNPs) in the *IL17R* and *IL21R* genes and assessed their associations with T1D susceptibility and with the presence of islet and extra-pancreatic autoantibodies.

## Materials and methods

All procedures were conducted in accordance with the Declaration of Helsinki. Approval of the Ethical Committee of the Hospital das Clínicas da Faculdade de Medicina da Universidade de São Paulo (CAPPesq CAAE 02038312.1.0000.0068) and written consent from patients or parents were obtained before research procedures.

All serum and plasma samples were collected in fasting conditions, according to the kit manufacturers’ guidelines. The samples were separated by centrifugation and stored at -80°C and the dosages were promptly performed.

**Figure 1:**
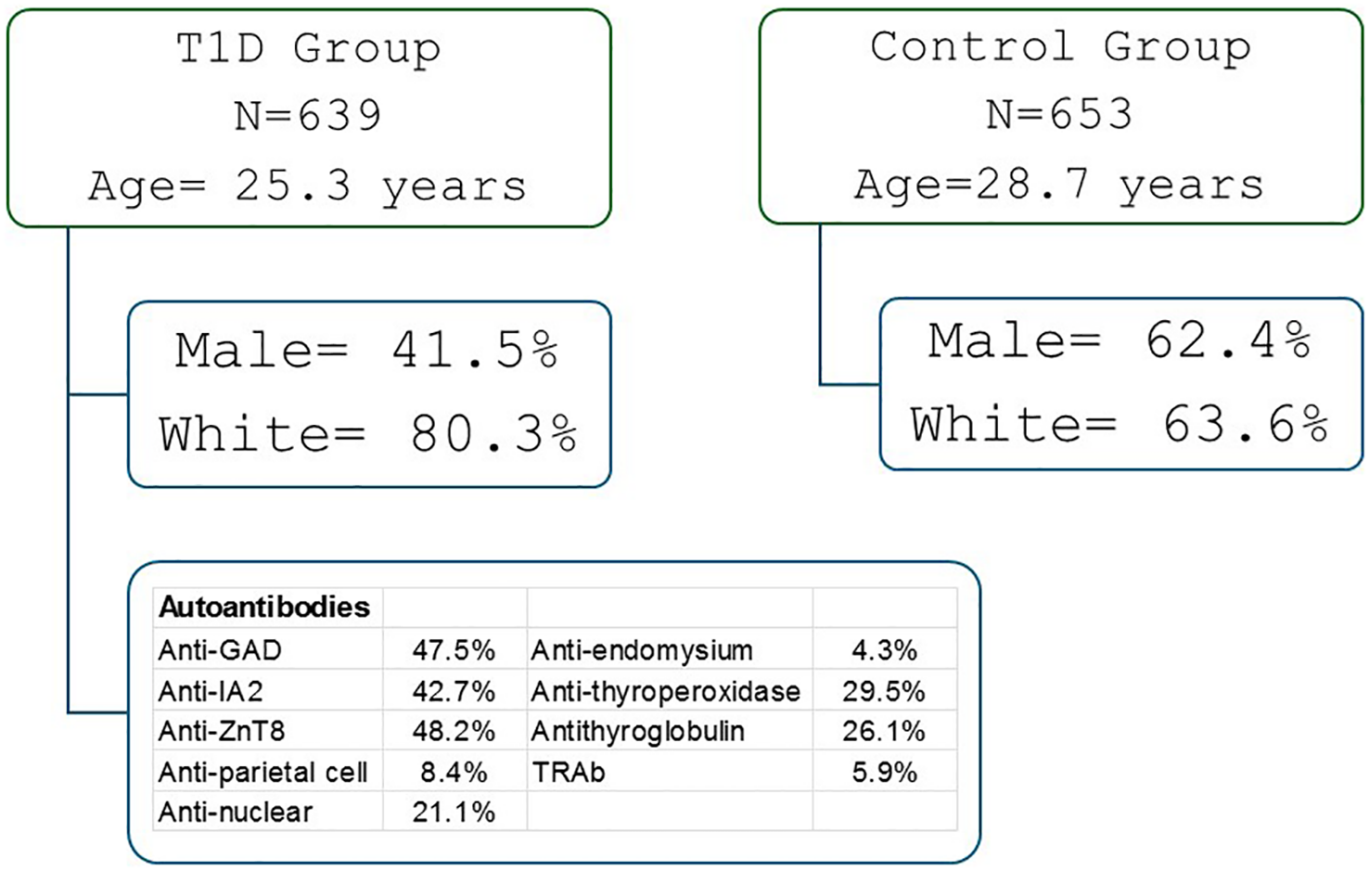
Demographic characteristics of patents with type 1 diabetes (T1D) and health controls.

This cross-sectional genotyping study included 639 patients with T1D, attended at the Clinical Hospital and 653 health controls (according to ADA criteria) (27). The inclusion criteria for T1D patients were persistent insulin requirement and the presence of one or more islet autoantibodies. The exclusion criteria considered other types of diabetes (T2D, mature onset diabetes of the young and secondary diabetes) and use of medications except insulin. The control group was composed of healthy people from the army, scouts or college students who agreed to participate in the study. Included islet autoantibody negative individuals with normal fasting glucose and Hb1Ac levels, and no personal or family history of autoimmune diseases (**Figure 1**).

The demographic and clinical characteristics of the participants are presented in **Supplementary Table 1**. Compared to controls, T1D patients were younger (25.3 ± 112.8 *vs*. 28.7 ± 11.6 years), had higher blood glucose levels(192.1 ± 115.8 x 83.6 ± 9.8mg/dL), a higher proportion of females (58.5 x 37.6%), a greater frequency of pancreatic and extra-pancreatic autoantibodies, and a higher proportion of self-reported white skin color (80.3 x 63.6%) (p < 0.001). The mean age at diagnosis was 11.9 ± 7.8 years, and the mean disease duration was 13.2 ± 10.9 years. Approximately 116 patients with T1D were treated with thyroid hormone. One patient had celiac disease, and one had atrophic gastritis.

The expression of IL-21 receptor in CD3+, CD4+ and CD8+ T cells was evaluated by flow cytometry assay in 35 patients (16 females/19 males) aged 8.4 ± 4.4 years with recent-onset T1DM (<6 months of diagnosis) and 25 health controls (12 females/13 males) aged 8.3± 4.1 years. The inclusion and exclusion criteria were the same as those for the genotyping study.

### Selection of Single Nucleotide Polymorphisms (SNPs)

Single nucleotide polymorphisms (SNPs) in the *IL17RA* and *IL21R* genes were selected from the HapMap database (http://hapmap.ncbi.nlm.nih.gov/) based on two populations, considering the high degree of miscegenation in the Brazilian population (32): CEPH (Utah residents with Northern and Western European ancestry; CEU) and Yoruba in Ibadan, Nigeria (YRI).

HapMap data were analyzed using Haploview software (www.broad.mit.edu/personal/cbaret/haploview/), a bioinformatics tool used to identify linkage disequilibrium (LD) and determine the “tag” SNPs representing the genetic variability of each LD block in the studied genes (29, 30).

Selected tag SNPs had a minor allele frequency greater than 10% and were common to both the CEU and YRI populations.

### Molecular study and biochemical evaluation

Genomic DNA was extracted from fresh peripheral blood cells using a conventional salting-out method (31). Variants in the *IL17RA* and *IL21R* genes were genotyped using the GoldenGate Platform with Veracode technology (Illumina, USA), according to the manufacturer’s instructions.

Autoantibodies were measured as follows: anti-glutamic acid decarboxylase (anti-GAD65), anti-tyrosine phosphatase (anti-IA2), anti-thyroid peroxidase (anti-TPO), and anti-thyroglobulin (anti-TG) by radioimmunoassay; anti-zinc transporter 8 (anti-ZnT8) by ELISA; anti-parietal cell antibody by direct immunofluorescence; and anti-TSH receptor antibodies (TRAb) by radioreceptor assay.

### Expression of IL-21 receptor analysis by flow cytometry assay

Peripheral mononuclear blood cells (PMBCs) were separated from blood by centrifugation over Ficoll-Hypaque gradient. PMBCs were washed twice with the staining buffer, phosphate-buffered saline (PBS) containing 2% fetal bovine serum. Cells (5 × 10^5^) were stained on ice for 20 min with 10 µL of the following monoclonal antibodies: phycoerythrin-conjugated mouse anti-human IL-21R mAB (R&D systems, Minneapolis, MN), fluorescein isothiocyanate (FITC)-conjugated mouse anti-human CD3 (Beckman Coulter, Fullerton, CA), ECD-conjugated mouse anti-human CD4 (Beckman Coulter, Fullerton, CA), and PC5-conjugated mouse anti-human CD8 (Beckman Coulter, Fullerton, CA). Stained cells were washed twice with 2 mL of PBS-azide for 2 min at 500 g. The cellular button was resuspended in 400 µL of 4% PBS-paraformaldehyde and then analyzed using a Coulter Epics XL-MCL flow cytometer and Summit v 4.0 software (DakoCytomation, USA). We acquired 10.000 events for the analysis of the receptor. The expression of the receptor was analyzed considering the percentage of CD3+, CD4+ and CD8+ T cells expressing the receptor.

### Statistical analysis

The polymorphic allele was defined as the least frequent allele in the study population and referred to as the rare allele. The distribution of variables was assessed using the Kolmogorov-Smirnov test. Parametric and non-parametric numerical variables were analyzed using Student’s *t*-test and Mann-Whitney U test, respectively. Qualitative variables were compared using the chi-square test or Fisher’s exact test, with SPSS^®^ version 18.0. Associations were evaluated under three genetic models: genotypic, dominant, and recessive. Analyses were adjusted for covariates including sex, age, and self-reported skin color when assessing T1D risk. Associations with autoantibody positivity were adjusted for sex, duration of diabetes, and self-reported skin color. A *p*-value < 0.05 was considered statistically significant.

This study was exploratory in nature. So, a convenience sample was used, in which we included all patients diagnosed with T1D seen at the outpatient clinic. The control group was composed of healthy people from the army, scouts or college students who agreed to participate in the study. At any event, taking into account 639 T1D patients and 653 controls the study was adequately powered to detect polymorphisms with minimal allele frequency of 0.1, relative risk of 1.65, alpha 0.05, and power >0.8.

## Results

SNPs in *IL17RA* and *IL21R* were in Hardy-Weinberg Equilibrium (HWE) in both groups (**Supplementary Table 2**). Rare allele frequencies were similar to those observed in the CEU population (**Supplementary Table 3**).

Two *IL17RA* variants were associated with T1D and remained statistically significant after adjustment for covariates. The rare G allele of rs2241049 conferred a 1.42-fold increased risk for T1D (*p* adj = 0.005), while the AA genotype of rs879577 was implicated in protection against T1D (OR adj = 0.61, *p*adj = 0.021*) (**Table 1**). Furthermore, the A allele of rs879577 and the GG genotype of rs5748863 were associated with a lower frequency of anti-IA2 autoantibodies (**Table 2**). No *IL17RA* variants were involved with extra-pancreatic autoantibodies positivity (**Supplementary Table 4**).

**Table 1:** Genotypic frequencies of the variants in *IL-17RA and IL-21R* in patients with T1D and control subjects and their association with T1D susceptibility.

Genes	Variants	Genotypes	Controls (N%)	T1D (N%)	OR	CI 95%	*p*	OR adj	CI 95% adj	*p adj*
***IL17RA***	rs5748863	AG+GG	465 (72.7)	431 (69.2)	0.845	(0.662 - 1.077)	0.174	0.93	(0.72 - 1.2)	0.59
		GG	127 (19.8)	125 (20.1)	1.014	(0.769 - 1.336)	0.922	1.12	(0.84 - 1.51)	0.433
	rs2241049	AG+GG	357 (56.2)	379 (63.3)	1.342	(1.068 - 1.686)	0.012*	1.42	(1.11 - 1.81)	0.005*
		GG	85 (13.4)	94 (15.7)	1.204	(0.877 - 1.654)	0.25	1.22	(0.87 - 1.7)	0.259
	rs879577	AG+AA	322 (52.2)	302 (49.3)	0.89	(0.711 - 1.113)	0.305	0.96	(0.76 - 1.21)	0.703
		AA	72 (11.7)	42 (6.9)	0.557	(0.374 - 0.829)	0.004	0.61	(0.4 - 0.93)	0.021*
	rs5992628	TG+TT	439 (69.8)	420 (68.6)	0.947	(0.744 - 1.205)	0.656	0.9	(0.7 - 1.16)	0.426
		TT	110 (17.5)	134 (21.9)	1.323	(0.999 - 1.752)	0.051	1.26	(0.94 - 1.7)	0.12
**IL-21R**	rs2214537	CG+GG	403 (64.9)	370 (62.7)	0.91	(0.72 - 1.15)	0.429	0.95	(0.74 - 1.21)	0.665
		GG	99 (15.9)	90 (15.3)	0.949	(0.696 - 1.295)	0.742	1.02	(0.73 - 1.41)	0.928
	rs7199138	GC+CC	325 (51.3)	345 (56.5)	1.229	(0.983 - 1.537)	0.07	1.33	(1.05 - 1.68)	0.018*
		CC	63 (10)	64 (10.CV5)	1.059	(0.733 - 1.528)	0.761	1.08	(0.73 - 1.59)	0.693
	rs3093315	TG+TT	389 (60.4)	357 (57.3)	0.88	(0.703 - 1.101)	0.262	0.84	(0.67 - 1.07)	0.158
		TT	77 (12)	81 (13)	1.1	(0.788 - 1.536)	0.574	1.08	(0.76 - 1.54)	0.662
	rs2285452	AG+AA	308 (48.7)	300 (48.9)	1.008	(0.807 - 1.259)	0.942	1.02	(0.81 - 1.29)	0.851
		AA	52 (8.2)	49 (8)	0.969	(0.645 - 1.456)	0.88	1.01	(0.66 - 1.56)	0.958

T1D, type 1 diabetes patients; OR, odds ratio; CI, confidence interval; adj, adjusted for age gender and ancestry.

Bold values refer to significant statistical P value.

**Table 2:** Associations of *IL-17RA* variants with pancreatic autoantibodies in patients with type 1 diabetes.

IL-17RA	Genotypes	Anti-GAD65	*p*	Anti-Znt8	*p*	Anti-IA2	*p*	OR adj	IC (95%)	*p adj*
Variants		N (%)		N (%)		N(%)				
rs5748863	AA	79 (53.0)	0.168	41 (42.2)	0.165	62 (44.3)	0.013*	0.56	(0.32 - 0.99)	0.047*
	AG	123 (46.8)		86 (53.4)		116 (47.0)				
	GG	45 (41.3)		35 (44.3)		32 (30.2)				
	AA/AG					178(46%)				
	GG					32 (30.2)	0.004*	0.52	(0.32 - 0.86)	0.010*
rs2241049										
	AA	97 (52.4)	0.212	56 (47.0)	0.978	77 (43.0)	0.919			
	AG	109 (46.3)		72 (48.3)		93 (42.0)				
	GG	34 (41.4)		26 (48.1)		31 (40.2)				
rs879577										
	GG	122 (47.8)	0.976	80 (49.3)	0.934	113 (47.1)	0.053			
	AG	107 (47.8)		70 (47.2)		80 (37.2)				
	AA	17 (45.9)		11 (47.8)		11 (32.2)				
	GG					113 (47.0)				
	AG/AA					91 (36.6)	0.018*	0.67	(0.45 - 0.99)	0.043*
rs5992628										
	GG	73 (47.0)	0.581	49 (45.3)	0.619	60 (40.2)	0.2			
	TG	113 (47.0)		69 (47.5)		91 (40.4)				
	TT	61 (52.5)		41 (52.5)		55 (50.0)				

anti-GAD65, Islet Autoantibodies: anti- glutamic acid decarboxylase; anti-IA2, anti-tyrosine phosphatase; Anti-ZnT8, anti-zinc transporter 8.

Bold values refer to significant statistical P value.

Among *IL21R* variants, only rs7199138 (C allele) conferred increased susceptibility to T1D (OR adj = 1.33, *p* adj = 0.018*) (**Table 3**). No *IL21R* variants influenced islet autoantibody positivity (**Supplementary Table 5**), but associations with extra-pancreatic autoantibodies were observed (**Table 4**). The rs2214537 G allele was related to a lower frequency of anti-parietal cell antibodies (CG+GG: 4.9% *vs*. CC: 16.9%, *p* < 0.001), and the rs2285452 A allele to a lower frequency of anti-endomysium antibodies (AG+AA: 1.3% *vs*. GG: 7.3%; *p* adj = 0.025). Conversely, the rs2285452 AA genotype was associated with a higher frequency of anti-thyroid peroxidase antibodies (AA: 48.4% *vs*. GG+AG: 27.2%, ORadj = 2.38; *p* adj = 0.028), and the rs3093315 T allele with TRAb positivity (ORadj = 5.90, *p* adj = 0.024). In agreement with these data, the frequency of patients with T1D treated with thyroid hormone (levothyroxine) was higher in those carrying rs22285452A (22.16% x 16.63%; OR=1.427; p=0.0272). A similar trend was observed for those carrying rs3093315T (21.49% x 17.08%; OR=1.329; p= 0.058) (**Supplementary Table 6**).

**Table 3:** Genotypic frequencies of the variants in *IL-21R* in patients with T1D and control subjects and their association with T1D susceptibility.

Variant	Genotypes	Controls	T1D	OR	CI 95%	*p*	OR adj	CI 95% adj	*p adj*
		N (%)	N (%)						
rs2214537	CG+GG	403 (64.9)	370 (62.7)	0.91	(0.72 - 1.15)	0.429	0.95	(0.74 - 1.21)	0.665
	GG	99 (15.9)	90 (15.3)	0.949	(0.696 - 1.295)	0.742	1.02	(0.73 - 1.41)	0.928
rs7199138	GC+CC	325 (51.3)	345 (56.5)	1.229	(0.983 - 1.537)	0.07	1.33	(1.05 - 1.68)	0.018*
	CC	63 (10)	64 (10.5)	1.059	(0.733 - 1.528)	0.761	1.08	(0.73 - 1.59)	0.693
rs3093315	TG+TT	389 (60.4)	357 (57.3)	0.88	(0.703 - 1.101)	0.262	0.84	(0.67 - 1.07)	0.158
	TT	77 (12)	81 (13)	1.1	(0.788 - 1.536)	0.574	1.08	(0.76 - 1.54)	0.662
rs2285452	AG+AA	308 (48.7)	300 (48.9)	1.008	(0.807 - 1.259)	0.942	1.02	(0.81 - 1.29)	0.851
	AA	52 (8.2)	49 (8)	0.969	(0.645 - 1.456)	0.88	1.01	(0.66 - 1.56)	0.958

T1D, type 1 diabetes patients; OR, odds ratio; CI, confidence interval; adj, adjusted for age gender and ancestry.

Bold values refer to significant statistical P value.

**Table 4:** Associations of IL-21R variants with extra-pancreatic autoantibodies in patients with type 1 diabetes.

IL-21R Variants	Genotypes	Autoantibodies	Positive	Negative	*p*	OR adj	CI (95%) _adj_	*p adj*
			N(%)	N(%)				
rs2214537	CC	Anti- parietal cell	15 (16.9)	74 (83.1)	0.002*	0.24	(0.09 - 059)	<0.001*
	CG/GG		8 (4.9)	156 (95.1)				
rs3093315	GG	TRAb	2 (1.9)	105 (98.1)	0.016*	5.89	(1.26 - 27.61)	0.024*
	TG/TT		13 (9.2)	128 (90.8)				
rs2285452	GG	Ant-endomysium	11 (7.3)	139 (92.7)	0.02*	0.17	(0.04 - 0.8)	0.025*
	AG/AA		2 (1.3)	147 (98.7)				
rs2285452	GG/AG	Anti-TPO	97 (27.2)	260 (78.8)	0.012*	2.38	(1.1 - 5.13)	0.028*
	AA		15 (48.4)	16 (51.6)				

OR, odds ratio, CI, confidence interval, adj, adjusted for gender, self-reported color and duration of diabetes.

TRAb- Thyroid Stimulating Hormone autoantibody.

Bold values refer to significant statistical P value.

A trend toward a higher frequency of anti-thyroglobulin antibodies was associated with rs5992628G (*p*adj=0.073),

The expression of IL-21R (median) was reduced in patients with T1D both in CD3+ (27.4 x 35.0%; *p*=0.018) and in CD4+ T cells (10.02 x 17.0%; *p*=0.0028) but similar in CD8+T cells (16.5 x 15.7%; *p*=0.899) in comparison with controls (**Supplementary Tables 6, 7**).

## Discussion

Our findings indicate reduced expression of IL21R on peripheral CD4+ T cells in recent-onset T1D patients, which is in line with the reduced surface expression of IL-17RA previously observed by our group (14). In addition, findings suggest that genetic variants in the *IL17RA* and *IL21R* genes are associated with the development of Type 1 Diabetes (T1D) and the production of pancreatic and extra-pancreatic autoantibodies. The observation that the frequencies of the rare alleles closely resemble those of the CEU population aligns with previous studies in the Brazilian population, which reported European ancestry in 77% of T1D patients and 71% of controls (28) (**Supplementary Table 3**).

Two variants of the *IL17RA* gene were associated with T1D. Individuals carrying the G allele of the intronic rs2241049 variant exhibited a 1.42-fold increased risk of developing T1D. Polymorphisms in non protein-coding, regulatory regions of a given gene may disrupt transcription factor binding sites, altering its transcription and potentially affecting immune system homeostasis. Notably, previous studies reported the loss of binding sites for transcription factor FOXP3, a key regulator of regulatory T cell development (32), for C/EBPβ (33, 34), involved in inflammatory gene regulation including IL-17, and NF-Y, which influences gene expression through DNA structure modulation (35). However, the functional implications of the IL17RA rs2241049 variant remain unclear. While this polymorphism conferred protection to psoriasis, a Th17-driven disease successfully treated with anti-IL-17 monoclonal antibodies (36), it has been associated with increased severity in ulcerative colitis (37), primary lung graft dysfunction and neutrophilia, indicating a potential role in inflammatory responses (38).

Conversely, other IL17RA variants appeared protective. The AA genotype of the missense variant rs879577 is in exon 13 of the IL17RA gene and changes the amino acid sequence at position 367 from alanine to valine. It was associated with a reduced risk of T1D (ORadj = 0.61, padj = 0.021) and it is likely benign according to SIFT and Polyphen. However, we cannot rule out functional effects considering that the two amino acids have structural and biochemical differences. Furthermore, a variant in a coding region may also influence the expression levels of its gene; predictive analyses indicated that the rs879577AA variant may alter the binding site for the transcription factor YY1, which can act as both a repressor and activator of gene transcription (39).This variant also seems to protect against Alopecia Areata (40) and asthma (41). Both rs879577AA and the intronic rs5748863GG variants were linked to a lower frequency of anti-IA2 autoantibodies. Reduced levels of anti-IA2 have been associated with decreased IL-17 production (42). The IL17RA rs5748863G is an intronic variant with no known effect which does not appear to affect transcription factor binding sites. Nonetheless, the trend toward a higher frequency of anti-thyroglobulin antibodies associated with rs5992628G (p=0.073), a 3’UTR variant with no known effect, and previous reports linking the rs4819554G to decreased anti-GAD titers and reduced T1D risk (14) further support the role of IL17RA in autoimmunity. However, 3’UTR variants may hypothetically modify seed sequence binding of microRNAs, which are negative regulators of gene/protein expression. Our data imply that IL17RA polymorphisms affecting transcriptional regulation may influence T1D risk, potentially through epigenetic mechanisms governing gene expression plasticity. The expression of IL-17 signaling apparatus and of IL-17 receptors in islets might influence T1D progression (43).

The rs7199138 C allele of the *IL21R* gene was also associated with increased T1D susceptibility (OR = 1.33, *p*adj = 0.018). This variant may create a binding site for FOXP3, a negative regulator of the immune system, while disrupting the c-Ets1 binding site, which is crucial for cytokine and chemokine gene expression and lymphoid cell function. Ets1 deficiency in T cells has been linked to SLE-like autoimmunity in mice (44). Additionally, *IL21R* polymorphisms were associated with lower frequencies of autoantibodies against non-pancreatic antigens, such as parietal cells (rs2214537G: 4.9% *vs*. 16.9%, p < 0.001) and endomysium (rs2285452A: 1.3% *vs*. 7.3%, *p*adj = 0.025). Variants also influenced autoimmune thyroid disease markers, with higher frequencies of TRAb (rs3093315T: 9.2% *vs*. 1.9%, padj = 0.024) and thyroperoxidase antibodies (rs2285452A: 48.4% *vs*. 27.2%; padj = 0.028) in T1D patients. The rs2285452A variant has been previously associated with Hashimoto’s thyroiditis in individuals without diabetes (7). However, it seems not to alter binding sites for transcription factors and the mechanisms causing predisposition to autoimmunity are still unclear. In addition, an association between polymorphisms of the IL-21R with diabetes in Japanese patients (45), and with SLE in a European-derived cohort were documented (9). IL-21 is a cytokine with diverse and sometimes opposing effects (46, 47). It can promote IFN-γ and T-bet expression via STAT1 activation, enhancing antibody production, while also stimulating T and B cell proliferation and IL-10 production (a cytokine with immunosuppressive activity) through STAT3 activation. IL-21 can expand regulatory B cells (B10 cells), which suppress T cell responses, and induce apoptosis in B cells. Its effects on dendritic cells include both inhibition of maturation and promotion of IL-1β production. Other suppressive effects include the expansion of suppressor CD8 + T cells and the induction of IL-22 expression in CD4+ T cells. These varied actions may suggest that IL-21’s role in autoimmunity may depend on the cytokine milieu and *IL21R* variants.

Although our analyses of extra-pancreatic autoantibodies were performed only in patients with T1D, the associations of the IL-21R and IL-17RA variants with T1D and autoantibodies targeting other nonpancreatic organs are probably due to extensive overlap in pathogenic mechanisms shared by different autoimmune disorders. A shared genetic background seems to confer predisposition across several autoimmune diseases. The reduced expression of IL17RA (14) and of IL21R on peripheral T cells in recent-onset T1D patients observed by our group may result from negative transcriptional regulation associated with the IL17RA and IL21R polymorphisms described here. Decreased expression of IL- 21RA on peripheral B lymphocytes (46) was also observed in another autoimmune disease - SLE, associated with nephritis and high-titer anti-double-stranded DNA antibodies (48). However, we cannot exclude that the observed reduced surface expression may be due to receptor internalization following cytokine engagement, a process observed with other receptors like IL-7R (49). Factors such as receptor trafficking, glycosylation changes due to hyperglycemia (50), or differences between peripheral and tissue-specific immune environments may also contribute.

Limitations of the study include the heterogeneity and genetic admixture that is characteristic of the Brazilian population. Analyses were adjusted for covariates including sex, age (or duration of diabetes) and self-reported skin color when assessing T1D risk or frequency of autoantibodies in order to minimize the influence of variables. A second limitation is the lack of replication in an independent population.

Our study demonstrated that genetic variants in *IL17RA* and *IL21R* were associated with T1D susceptibility, and that variants of IL-21R also predispose to the development of nonpancreatic thyroid- and gastrointestinal-specific autoantibodies. Specifically, *IL-17RA* variant rs879577, related to T1D protection and lower frequency of anti-IA2, may aid in the prevention of T1D and deserves more studies. These findings highlight the complex interplay between cytokine receptor polymorphisms and autoantibody/autoimmune disease phenotypes, emphasizing the multifactorial nature of T1D pathogenesis involving multiple genetic and immunological networks. Future directions include functional analysis of the variants in large populations to elucidate the precise mechanism by which they confer autoimmune risk or protection and longitudinal follow-up of patients carrying the associated variants as biomarkers both of T1D progression and severity and for the development of other autoimmune diseases.

## Conclusion

Our study demonstrated that genetic variants in *IL17RA* and *IL21R* were associated with T1D susceptibility and that variants of *IL-21R* also predispose to other autoimmune diseases affecting thyroid and gastrointestinal tract tissues. In special the missense mutation rs879577, related to T1D protection and lower frequency of anti-IA2 may provide more information about the pathogenesis of T1D. These findings highlight the complex interplay between cytokine receptor polymorphisms and autoimmune disease phenotypes, emphasizing the multifactorial nature of T1D pathogenesis involving multiple genetic and immunological networks.

Future directions include longitudinal follow-up of patients carrying the associated variants as biomarkers both of T1D progression and severity, as well as biomarkers for the development of other autoimmune diseases.

## Data Availability

The datasets presented in this study can be found in online repositories. The names of the repository/repositories and accession number(s) can be found in the article/**Supplementary Material**.
